# Dietary Patterns in Early Childhood and the Risk of Childhood Overweight: The GECKO Drenthe Birth Cohort

**DOI:** 10.3390/nu13062046

**Published:** 2021-06-15

**Authors:** Outi Sirkka, Maria Fleischmann, Marieke Abrahamse-Berkeveld, Jutka Halberstadt, Margreet R. Olthof, Jacob C. Seidell, Eva Corpeleijn

**Affiliations:** 1 Department of Health Sciences, Faculty of Science, Vrije Universiteit Amsterdam, Amsterdam Public Health Research Institute, 1081 HV Amsterdam, The Netherlands; m.s.fleischmann@vu.nl (M.F.); j.halberstadt@vu.nl (J.H.); margreet.olthof@vu.nl (M.R.O.); j.c.seidell@vu.nl (J.C.S.); 2 Danone Nutricia Research, 3584 CT Utrecht, The Netherlands; Marieke.Abrahamse@danone.com; 3 Department of Epidemiology, University of Groningen, University Medical Center Groningen, 9713 GZ Groningen, The Netherlands; e.corpeleijn@umcg.nl

**Keywords:** overweight, diet, dietary pattern, childhood

## Abstract

Limited and inconsistent evidence exists on the associations between dietary patterns and overweight during childhood. The present study describes dietary patterns of three-year-old Dutch children and associations between childhood overweight and body mass index (BMI) development between 3 and 10 years. In the GECKO Drenthe birth cohort (N = 1306), body height and weight were measured around the age of 3, 4, 5, and 10 years, and overweight was defined according to Cole and Lobstein. A validated food frequency questionnaire (FFQ) was used to measure diet at 3 years. Dietary patterns were derived using principal components analysis (PCA). Using logistic regression analyses, pattern scores were related to overweight at 3 and 10 years. A linear mixed-effect model was used to estimate BMI-SDS development between 3 to 10 years according to quartiles of adherence to the pattern scores. Two dietary patterns were identified: (1) ‘minimally processed foods’, indicating high intakes of vegetables/sauces/savory dishes, and (2) ‘ultra-processed foods’, indicating high intakes of white bread/crisps/sugary drinks. A 1 SD increase in the ‘ultra-processed foods’ pattern score increased the odds of overweight at 10 years (adjusted OR: 1.30; 95%CI: 1.08, 1.57; *p* = 0.006). The ‘minimally processed foods’ pattern was not associated with overweight. Although a high adherence to both dietary patterns was associated with a higher BMI-SDS up to 10 years of age, a stronger association for the ‘ultra-processed foods’ pattern was observed (*p* < 0.001). A dietary pattern high in energy-dense and low-fiber ultra-processed foods at 3 years is associated with overweight and a high BMI-SDS later in childhood.

## 1. Introduction

Childhood overweight and obesity are important public health challenges because they are associated with an increased risk of obesity later in life [1] as well as other adverse health consequences [2]. Increasing evidence suggests that unhealthy diets during early childhood are key modifiable risk factors for the development of overweight and obesity [3,4,5]. Improving dietary habits is thus one of the crucial strategies for the prevention of overweight.

Dietary patterns represent a comprehensive picture of the whole diet and may thus give a better indication of a disease risk than by looking at the consumption of individual foods or nutrients [6]. There is growing evidence for adults and older children that dietary patterns that are high in energy-dense, nutrient-poor, low-fiber foods predispose to later overweight and obesity [5,7]. However, studies in young children (2 to 5 years) are limited and have shown inconsistent findings. 

Most previous studies on dietary patterns including young children have identified two major dietary patterns. One pattern indicated a high intake of energy-dense, high-sugar/fat, low-fiber foods, often labeled as ‘unhealthy’ or ‘processed’ [8,9,10,11,12,13,14,15,16,17,18]. In cross-sectional studies among 3- to 7-year-old children, a high adherence to such patterns was associated with a higher risk of obesity [8,10,12]. However, cross-sectional studies including younger children reported no associations [9,11,13]. Another dietary pattern commonly identified across studies indicated a high intake of fruits and vegetables, cereals, and/or dairy products, named as ‘traditional’ [8,9,11] or ‘healthy’ [8,9,10,12,13]. Two cross-sectional studies [8,11] reported an association between a high adherence to these patterns and a lower risk for overweight or obesity, while others [9,10,13] did not. Interestingly, one study found that a higher adherence to a ‘traditional’ pattern was associated with a higher BMI [12]. 

Prospective studies have also shown inconsistent findings. Among 2- to 5-year-old children, three studies found that a high adherence to a ‘snacking’ or ‘energy-dense’ pattern or ‘processed foods’ cluster was associated with an increase in BMI [14,15] or excess fat mass [16]. However, another study showed no association between a ‘junk food’ pattern at 3 years and obesity at 7 years [18]. In one study, an association with BMI at 7 years was found only among girls [17]. Moreover, studies with the longest follow-up period (up to 5 years) reported either no or mixed associations between various dietary patterns at age 3 or 5 and weight-related outcomes later in childhood [18,19].

Based on the aforementioned findings, current evidence on the associations between dietary patterns of young children and the development of childhood overweight or obesity is conflicting. Direct comparison between studies is also difficult due to differences in methodology to derive dietary patterns (PCA, cluster analysis, or reduced rank regression), various outcome measures used, and the fact that dietary patterns are population-specific. Because dietary habits are established early and track into later life [20,21,22,23], and longer-term follow-up studies on this topic are lacking, further knowledge is crucial for developing prevention strategies for overweight and obesity. Therefore, the objective of this study was to (1) identify the dietary patterns of three-year-old Dutch children by using principal component analysis (PCA) and (2) examine associations between dietary patterns and overweight cross-sectionally at 3 years and prospectively at 10 years. Additionally, we examined associations between dietary patterns and BMI-SDS development between 3 and 10 years of age.

## 2. Materials and Methods

### 2.1. Subjects

Data were obtained from a Dutch population-based cohort study, embedded within the Groningen Expert Center for Kids with Obesity (GECKO), the GECKO Drenthe study. Details of the study design, recruitment, and study procedures are described in detail elsewhere [24]. All pregnant women living in the province of Drenthe, the Netherlands, were invited to participate in this study during the third trimester of their pregnancy. Of the 5326 children born between April 2006 and April 2007, 2842 started active participation in the study. At 10 years, 2299 children still participated in the study. For the identification of dietary patterns among the study population, all children with complete data on the food frequency questionnaire (FFQ) at 3 years were included (N = 1306). From these children, all children with complete data on the covariates and for whom height and weight measurements were obtained at 3 and 10 years were selected for the analyses on the dietary patterns and overweight (N = 938, Figure S1). The majority of missing questionnaires at the age of 3 years was related to logistical issues. Characteristics and available BMI measurements of the children excluded from the analyses (because of the missing data on growth measurements or covariates) were similar, except for the educational status of the mother, which was slightly lower among those excluded. For the analysis on the dietary patterns and BMI-SDS development between 3 and 10 years, children with complete data on FFQ, covariates, and at least one BMI measurement during the period of 3 to 10 years were included, N = 1233. Written informed consent was obtained from the parents. The study was conducted according to the guidelines of the Declaration of Helsinki and was approved by the Medical Ethics Committee of the University Medical Center Groningen (Medical ethical approval ID: 2005.260). The cohort is registered at www.birthcohorts.net (accessed on 10 May 2021).

### 2.2. Dietary Data

Information on the diets at 3 years was obtained through an FFQ, completed by the parents. This FFQ has been validated for energy intake against the doubly labelled water method in a group of 4- to 6-year-old children [25]. The FFQ assessed the frequency of weekly consumption of 71 food products over the previous four weeks. Answer categories ranged from ‘never’ to ‘6–7 days a week’. For 27 food products, additional questions were included regarding the type or brand of the product consumed. Portion size was assessed using fixed units (e.g., slices of bread) or common household measures (e.g., cups, spoons). Parents were asked to measure the volume of glasses and cups used for different beverages. From these data, intake of each food product (in grams per day) was calculated. Daily energy and nutrient intakes were calculated based on the Dutch food composition database (2011) [26]. For the purpose of the current analysis, food intake data were collapsed into 33 food groups taking into account the nutrient composition, i.e., energy density, fat, and fiber content, as well as the usage of each food item. These 33 food groups are listed in Table S1.

### 2.3. Growth Measures during Childhood

The weight and height of the children were measured at ages 3, 4, 5, and 10 years during routine health examinations by the staff of the Youth Health Care centers (YHC). Body weight was measured using an electronic scale and rounded to the nearest 0.1 kg. Height was measured in the standing position against a wall and rounded to the nearest 0.1 cm. For the analyses on overweight at 3 and 10 years, age- and sex-specific BMI standard deviation scores (SDS) were derived using the WHO growth references [27,28] using the Growth Analyzer Software, version 4.0 (Growth Analyzer BV). Because the number of children with obesity (n = 17 and n = 39 at 3 and 10 years, respectively) were too small to be analyzed separately, the outcome was defined as overweight including obesity (referred to as overweight in the Methods and Results sections) and defined according to Cole and Lobstein [29]. To investigate the BMI-SDS development between 3 and 10 years, all BMI measurements at ages 3, 4, 5, and 10 years were included. These were calculated according to the age- and sex-specific distributions of BMI derived from the Dutch growth reference (2010) [30]. This reference was selected as it covers the period of 0–21 years, (thus including the period between 3 and 10 years), whereas the WHO provides two separate standards for the periods of 0–5 [27] and 5–19 years [28].

### 2.4. Covariates

Maternal covariates included pre-pregnancy BMI, highest completed educational level, ethnicity, smoking during pregnancy (yes/no), age (continuous, years), and parity (primiparous/multiparous) [31,32,33]. The information on the maternal covariates was collected during the last trimester of pregnancy or shortly after birth by means of a questionnaire and maternity files. Pre-pregnancy BMI was calculated from the weight and height data and dichotomized as normal weight (BMI < 25 kg/m^2^) or overweight (≥25 kg/m^2^). Educational level was categorized as low (no education, primary school, lower vocational, or lower general secondary education), middle (intermediate vocational training or higher secondary education) or high (higher vocational or university education) [34]. Ethnicity was defined as the country of birth of the mother and dichotomized as Dutch (mother being born in the Netherlands) or non-Dutch [35]. Child covariates included gestational age (weeks) and birth weight (g), which was rounded to the nearest 5 g. These data were reported by the midwives and obtained through the YHC registry. Birth weight was standardized according to gestational age- and sex-specific reference values based on the WHO reference [36]. These were derived using the Growth Analyzer Software, version 4.0 (Growth Analyzer BV). For 59 children, data on at least one of the covariates was missing. Due to the low number of missing data on the confounders, we decided not to impute missing data.

### 2.5. Statistical Analysis

Principal component analysis (PCA) with varimax rotation was used to identify dietary patterns among the total study population with valid dietary data (n = 1306). The number of patterns was selected considering eigenvalues of >1.50, the scree plot, and the interpretability of the patterns. We identified two dietary pattern components that had factor loadings >2 (2.76 and 2.02) and explained 14.5% of the variance in food intake. In the scree plot, a clear break was indicated after the second component, suggesting a two-component solution. The interpretability of the components was preferred over the second-best option, a five-component solution, which explained 29.6% of the total variance. Regarding the interpretability, the disadvantage of the five-component solution was that two components depicted meals rather than a diet (e.g., ‘breakfast’ with a high intake of food groups such as bread, butter/oil, and sweet bread toppings). For the two identified dietary patterns, regression-based factor scores were created by summing the observed standardized consumption per food group, which were weighted according to the PCA loadings. These factor scores were derived for each child and indicated the adherence to each dietary pattern. 

Logistic regression analyses were used to study the associations between the dietary pattern scores (continuous variable) and overweight at 3 and 10 years (N = 938). First, we carried out an analysis to estimate cross-sectional associations between the dietary patterns and overweight at 3 years. Then, we analyzed prospective associations of the dietary patterns and overweight at 10 years. In both analyses, the pattern scores for both dietary patterns were used as determinants simultaneously in order to adjust for the adherence to the other pattern. At both ages, we estimated two models; a crude model including the dietary pattern scores and a second adjusted model including the a priori selected child and maternal covariates. As sensitivity analyses, we also repeated the logistic regression analyses using the definition of overweight according to WHO (Tables S2 and S3). According to this definition, overweight is defined as +2 BMI-SDS at 3 years [27] and +1 BMI-SDS at 10 years [28].

To assess the association between dietary pattern scores and BMI-SDS development between 3 to 10 years, a random effects linear regression model with robust standard errors, to correct for the nesting (several measurements per child), was used. These analyses included all children who had at least one BMI measurements available between 3 to 10 years (N = 1233, see Tables S4 and S5). On average, each child had 3.3 BMI measurements (min 1, max 4). We estimated one model including both dietary pattern scores and interactions between the dietary patterns and age (categorical: 3, 4, 5, and 10 years) to account for non-linearity between age and BMI-SDS development. This model was adjusted for all covariates.

Descriptive data analysis and the logistic regression analyses were conducted using the statistical program SPSS, version 19 (Chicago, IL, USA). The random effects linear regression model was conducted using Stata, version 16 (StataCorp. 2019. Stata Statistical Software: Release 16. College Station, TX, USA: StataCorp LLC).

## 3. Results

The mothers of the children in the study population were mainly Dutch, 28.6% had a low educational level, and 38.5% had overweight (Table 1). The percentage of children with overweight (according to Cole and Lobstein) at 3 and 10 years were 13.6% and 16.4%, respectively. 

Table 2 shows the two dietary patterns identified, their factor loadings, and correlations with nutrients. The two patterns accounted for 14.5% of the variance in food consumption within our study population. For ease of description, we called these patterns ‘minimally processed foods’ and ‘ultra-processed foods’. The names were based on the characteristics of the foods that distinguished the two patterns. The first pattern ‘minimally processed foods’ was characterized by high intakes of vegetables, sauces, rice/pasta, and savory dishes. The second pattern, ‘ultra-processed foods’, indicated high intakes of white bread, crisps, savory snacks, and sugar-sweetened beverages (SSB), as well as low intakes of whole-grain bread. 

### 3.1. Cross-Sectional and Prospective Associations between Dietary Patterns and Overweight at 3 and 10 Years

At 3 years, no associations between the two dietary patterns and overweight were observed (Table 3). At 10 years (Table 4), a 1 SD higher score for the ‘ultra-processed foods’ pattern was positively associated with 1.36 times (95% CI: 1.14, 1.61) higher odds of overweight (crude model). This association remained statistically significant after adjusting for confounders (OR 1.30; 95% CI: 1.08, 1.57). Results from the sensitivity analyses including overweight definition according to the WHO were comparable to the main results at both ages (Tables S2 and S3). 

### 3.2. BMI-SDS Development

**Figure 1:**
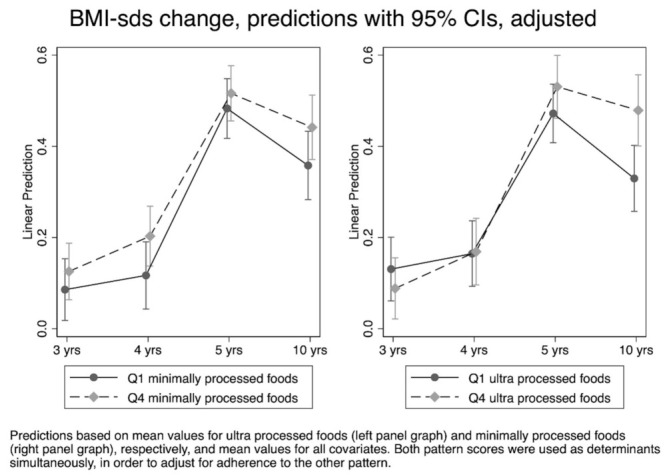
Linear predicted values for BMI-SDS development between 3 and 10 years of age, adjusted predictions with 95% CIs (N = 1233).

Children with a high adherence (Quartile 4) to the ‘minimally processed foods’ pattern showed higher BMI-SDS development between 3 and 10 years compared to those with a low (Quartile 1) adherence (Figure 1, left panel). The differences were significant at 4 and 10 years (*p* < 0.05; Table S4). Children with a high adherence (Quartile 4) to the ‘ultra-processed foods’ pattern had a BMI development towards a higher BMI-SDS from 5 years onwards, than children with a low adherence (Quartile 1) to this pattern (Figure 1, right panel). This difference was statistically significant at 10 years (*p* < 0.001; Table S4).

## 4. Discussion

In this study, conducted in Dutch children aged 3 years at baseline, two dietary patterns, which we labelled as ‘minimally processed foods’ and ‘ultra-processed foods’, respectively, were identified. We observed that a greater adherence to the ‘ultra-processed foods’ pattern was associated with a statistically significant increase in the odds of overweight and development of a significantly higher BMI-SDS up to 10 years. No cross-sectional associations between the dietary patterns and overweight at 3 years were observed.

Most previous studies have identified a pattern featuring a mixture of nutrient-poor, low-fiber ultra-processed foods, often labeled either as ‘processed’ or ‘junk food’ pattern [7]. In our study, the ‘ultra-processed foods’ pattern was not cross-sectionally associated with overweight at 3 years, which is in line with some [9,11,13] but not all [8,10,12] previous cross-sectional studies in young children. One possible explanation for our finding is that until the age of 3, the diets of young children are still evolving due to the changes in parental feeding practices and/or child feeding behaviors (e.g., fussy eating) [37,38] and therefore, associations with overweight at this age may not yet be evident. However, cross-sectional studies, where dietary intake and weight status are assessed at the same time, do not allow us to make conclusions of the causality of the associations. For instance, if a child has overweight or obesity, parents may change the child’s diet in an effort to prevent further weight gain or encourage weight loss. As a result, associations reported in cross-sectional studies may reflect reverse causation and therefore should be interpreted with caution.

In contrast to the cross-sectional findings, our prospective findings on ‘ultra-processed foods’ and overweight development up to age 10 were in line with several previous prospective studies in children [14,15,16,17]. In these studies, a high adherence to a similar pattern was positively associated with the development of obesity, higher BMI [14], or other adiposity measures [14,16] during childhood. Previous prospective studies assessing dietary patterns among young children (2 to 5 years), showed in contrast to our results, that a ‘processed’ or ‘junk’ pattern was not associated with obesity [18], or that the association with BMI increase was observed only among girls [17]. One possible explanation why we, unlike the other studies, found an association between the ‘ultra-processed foods’ pattern and the development of overweight and BMI-SDS, may be that our study included a longer follow-up. The follow-up time in the other studies (2 to 3 years) might have been too short for obesity to have manifested at such a young age. Furthermore, some studies [16,39] suggested that a ‘junk food’ diet at a younger age may not lead to weight gain in the long term due to counter regulation of energy intake, because young children may compensate for high energy intakes from junk food at subsequent eating occasions. Our results suggest that this may not be the case as we showed that children as young as 3 years with a high adherence to an ultra-processed dietary pattern are at risk for developing overweight during childhood.

In the present study, the dietary patterns extracted with the PCA accounted for 14.5% of the total variance in food intake. This is in line with the findings of other studies in children [40,41,42]. The ‘minimally processed foods’ pattern identified in our study indicated a high consumption of fruits, vegetables, fish, and whole-grain bread, as endorsed by the Dutch dietary guidelines [43]. Nevertheless, this pattern also showed relatively high intakes of some (processed) foods considered less healthy, but traditionally consumed by Dutch children, such as fried potatoes, meat, sauces, and savory snacks. The ‘minimally processed foods’ pattern describes a combination of high factor loadings from two patterns described in previous studies: ‘traditional’ (meat, vegetables, and potatoes) [8,9,12] and ‘healthy’ (fruit, vegetables, and fish) [44]. In our study, this dietary pattern was not associated with overweight cross-sectionally or prospectively, which is in line with some [9,10] but not all [8,11] previous studies. However, we also observed that children with a high adherence to this pattern had a somewhat higher BMI-SDS at 4 and 10 years, but not at 5 years. Although we do not have a clear explanation for this finding, one other prospective study [44] found that a ‘healthy’ dietary pattern at 5 years was positively associated with weight development between 5 and 10 years.

A diet high in ultra-processed foods such as sugary drinks, crisps, and savory snacks can compromise healthy growth in different ways. These types of foods are high in added sugars and/or fats, contain low levels of fiber/nutrients, and have a high energy density [45]. In addition, such foods and beverages are also highly palatable and tend to be eaten at a fast rate [46]. A high palatability of the foods disrupts the innate appetite control [47], leading to greater energy consumption and weight gain [48]. Additionally, a fast eating rate in combination with a high energy density is suggested as a plausible mechanism by which increased consumption of highly palatable foods promotes higher body weight [49]. Part of the association between the ‘ultra-processed foods’ pattern and overweight may also be explained by the lack of fruits, vegetables, and other nutrient/fiber-rich foods, which was also evident in the ultra-processed pattern observed in our study. Our results suggest that a dietary pattern high in energy-dense, low-fiber ultra-processed foods during toddlerhood seems to be more strongly associated with the development of childhood overweight than a more healthy diet including whole foods [50].

This study has several strengths. The data analyzed come from a large population-based cohort, with a relatively high retention rate. The study population was representative of the educational level of the general Dutch population. Our study included a follow-up period of 7 years, with BMI measurements available at several timepoints. To the best of our knowledge, our study includes the longest follow-up so far among studies on dietary patterns in young children. For deriving the dietary patterns, we used a validated FFQ, which also assessed information on portion sizes. Additionally, the analyses were adjusted for several known confounders; however, information on sedentary and physical activity was not available. In the GECKO Drenthe cohort, objectively measured moderate-to-vigorous physical activity at 5–6 years of age was not associated with overweight [51].

Some limitations should also be acknowledged. First, compared to the full cohort population (ever active participation), the group of low educated mothers were less represented in the study population (due to missing FFQ data), which may have introduced some bias on educational level. However, the majority of the missing data on FFQ’s was due to logistical aspects and can be regarded as missing completely at random. Reassuringly, means of important weight-related outcomes and confounders of the included children were very comparable to the full cohort population. Second, because ethnic minorities were slightly underrepresented, our findings may therefore be less representative of the general Dutch population. Third, as in all dietary studies, recall bias and misreporting are potential sources of bias. Especially, food items high in sugar and fat, considered ‘unhealthy’, may have been underreported by the parent more often compared to ‘healthy’ foods [52]. This may have attenuated the association between the dietary patterns and the outcomes. Fourth, during the process of the PCA, certain decisions, such as the number of dietary patterns and the food group classification, are prone to subjectivity. Finally, as in all observational studies, we cannot rule out the possibility of residual confounding.

## 5. Conclusions

This study in Dutch children indicates that, at 3 years of age, a dietary pattern including high intakes of white bread, crisps, savory snacks, and sugary drinks is associated with a higher risk of overweight and a higher BMI-SDS later in childhood. In line with the current dietary guidelines, our results suggest that limiting the consumption of energy-dense and nutrient-poor ultra-processed foods during childhood may be important for the prevention of childhood overweight and obesity.

## Figures and Tables

**Table 1:** Characteristics of the study population (n=1306).

Maternal Characteristics	n (%) ^1^
Age, mean (SD)	31.2 (4.2)
Educational level	
Low	28.6 (368)
Middle	32.8 (422)
High	38.7 (498)
Ethnicity	
Dutch	97.5 (1259)
Other	2.5 (32)
Pre-pregnancy BMI, mean (SD)	24.8 (4.7)
Overweight % (n)	38.5 (494)
Parity	
Multiparous	39.6 (516)
Smoking during pregnancy	
Yes	11.7 (152)
**Child characteristics**	
Sex	
Male	50.5 (660)
Birth weight (g), mean (SD)	3563.6 (557.0)
Age at diet measurement (years), mean (SD)	3.1 (0.4)
Age at 3-year BMI measurement (years), mean (SD)	3.1 (0.1)
Age at 10-year BMI measurement (years), mean (SD)	10.6 (0.5)
BMI and overweight at 3 years (n = 938)	
BMI, mean (SD)	16.0 (1.2)
Overweight (%, n); according to Cole and Lobstein [29]	13.6 (128)
Overweight (%, n); according to WHO [27]	3.5 (33)
BMI and overweight at 10 years (n = 938)	
BMI, mean (SD)	17.8 (2.8)
Overweight (%, n); according to Cole and Lobstein [29]	16.4 (154)
Overweight (%, n); according to WHO [28]	22.3 (209)

^1^ All values % (n) unless otherwise indicated. Missing values: education (n = 18), smoking (n = 3), ethnicity (n = 15), pre-pregnancy BMI (n = 23), birth weight (n = 13), gestational age (n = 13), parity (n = 3), age of the mother (n = 3).

**Table 2:** Factor loadings for the identified dietary patterns.

	Factor Loadings	
	Dietary Pattern 1 ‘Minimally Processed Foods’	Dietary Pattern 2 ‘Ultra-Processed Foods’
Water	0.20	−0.04
Vegetables	0.61	−0.17
Fruit	0.18	−0.22
Whole-grain bread	0.28	−0.65
Fish	0.30	0.01
Sauces	0.58	0.00
Potatoes, plain	0.41	0.14
Eggs	0.27	0.09
Fried and baked potatoes	0.40	0.18
Savory dishes	0.48	−0.03
Chicken	0.32	0.03
Meat	0.43	0.29
Milk and buttermilk	0.12	−0.21
Dairy desserts	0.23	0.07
Crisps	0.26	0.41
Cheese	0.28	−0.18
Cakes and confectionery	0.22	0.26
Butter and oil	0.14	0.02
White bread	−0.15	0.64
Breakfast cereals	0.16	−0.12
Added sugar	0.16	0.17
Sweet bread toppings	0.05	0.05
Sugar-sweetened beverages	0.14	0.34
Cookies	0.24	0.27
Rice and pasta	0.53	−0.26
Vegetarian meat substitutes	0.02	−0.26
Porridge	0.11	0.02
Soya milk products	0.01	−0.11
Nuts and raisins	0.25	0.00
Crackers	0.12	0.00
Savory snacks	0.31	0.41
Dairy drinks with sugar	0.04	0.34
Light drinks	0.02	0.07
Nutrients	Pearson’s correlation coefficient	
Total energy, mean (kcal/d)	0.7 **	0.4 **
Protein (E%)	0.3 **	−0.2 **
Fat (E%)	0.2 **	0.1 *
Carbohydrates (E%)	−0.3 **	0.1 *
Mono- and disaccharides (E%)	−0.2 **	0.1 **
Fiber (g/MJ)	0.7 **	−0.1 **

Extraction Method: Principal Component Analysis. Rotation Method: Varimax with Kaiser Normalization. Rotation converged in three iterations. Factor loadings > ±0.3 highlighted. ** *p* < 0.01, * *p* < 0.05.

**Table 3:** Associations between dietary patterns and overweight at 3 years (N = 938).

	Overweight at 3 Years *					
	Model 1, Crude			Model 2, Adjusted		
	OR	95% CI	*p*-Value	OR	95% CI	*p*-Value
Dietary pattern						
Pattern 1: ‘minimally processed foods foods’	1.07	0.89, 1.28	0.46	1.10	0.91, 1.33	0.31
Pattern 2: ‘ultra-processed foods’	1.02	0.85, 1.23	0.67	0.94	0.77, 1.15	0.54

* defined as BMI-SDS > 1.310 for boys and >1.244 for girls according to Cole and Lobstein [29]. Both pattern scores were used as determinants simultaneously in order to adjust for the adherence for the other pattern. Model 2 is adjusted for maternal age, pre-pregnancy BMI, parity, ethnicity, maternal smoking during pregnancy, educational level, birth weight, and gestational age.

**Table 4:** Associations between dietary patterns and overweight at 10 years (N = 938).

	Overweight at 10 Years *					
	Model 1, Crude			Model 2, Adjusted		
	OR	95% CI	*p*-Value	OR	95% CI	*p*-Value
Dietary pattern						
Pattern 1: ‘minimally processed foods’	0.99	0.84, 1.18	0.94	1.03	0.86, 1.24	0.74
Pattern 2: ‘ultra-processed foods’	1.36	1.14, 1.61	0.001	1.30	1.08, 1.57	0.006

* defined as BMI-SDS > 1.310 for boys and >1.244 for girls according to Cole and Lobstein [29]. Both pattern scores were used as determinants simultaneously, in order to adjust for the adherence for the other pattern. Model 2 is adjusted for maternal age, pre-pregnancy BMI, parity, ethnicity, maternal smoking during pregnancy, educational level, birth weight and gestational age.

## Data Availability

The data presented in this study are available on request from E.C. (e.corpeleijn@umcg.nl). The data are not publicly available due to ethical reasons.

## Supplementary Materials

The following are available online at https://www.mdpi.com/article/10.3390/nu13062046/s1, Figure S1: Selection of the study population, Table S1: Food grouping for PCA, Table S2: Associations between dietary patterns and overweight at 3 years, sensitivity analysis using the WHO definition of overweight (N = 938), Table S3: Associations between dietary patterns and overweight at 10 years, sensitivity analysis using the WHO definition of overweight (N = 938). Table S4: Mean differences in estimated BMI-SDS for quartile of adherence (Q1 vs. Q4) to the dietary patterns at each age, derived from the random effects linear regression model (N = 1233), Table S5: Associations between dietary patterns and BMI-SDS development between 3 and 10 years of age (N = 1233).
