# Supplementary material for: Dietary Patterns in Early Childhood and the Risk of Childhood Overweight: The GECKO Drenthe Birth Cohort
Source: Nutrients. 2021 Jun 15;13(6):2046. doi: 10.3390/nu13062046 (PMC8232591; doi:10.3390/nu13062046)
Supplement: Supplementary file 1 [file nutrients-13-02046-s001.zip › nutrients-1238455-supplementary.pdf]

Supplementary Figure S1. Selection of the study population.

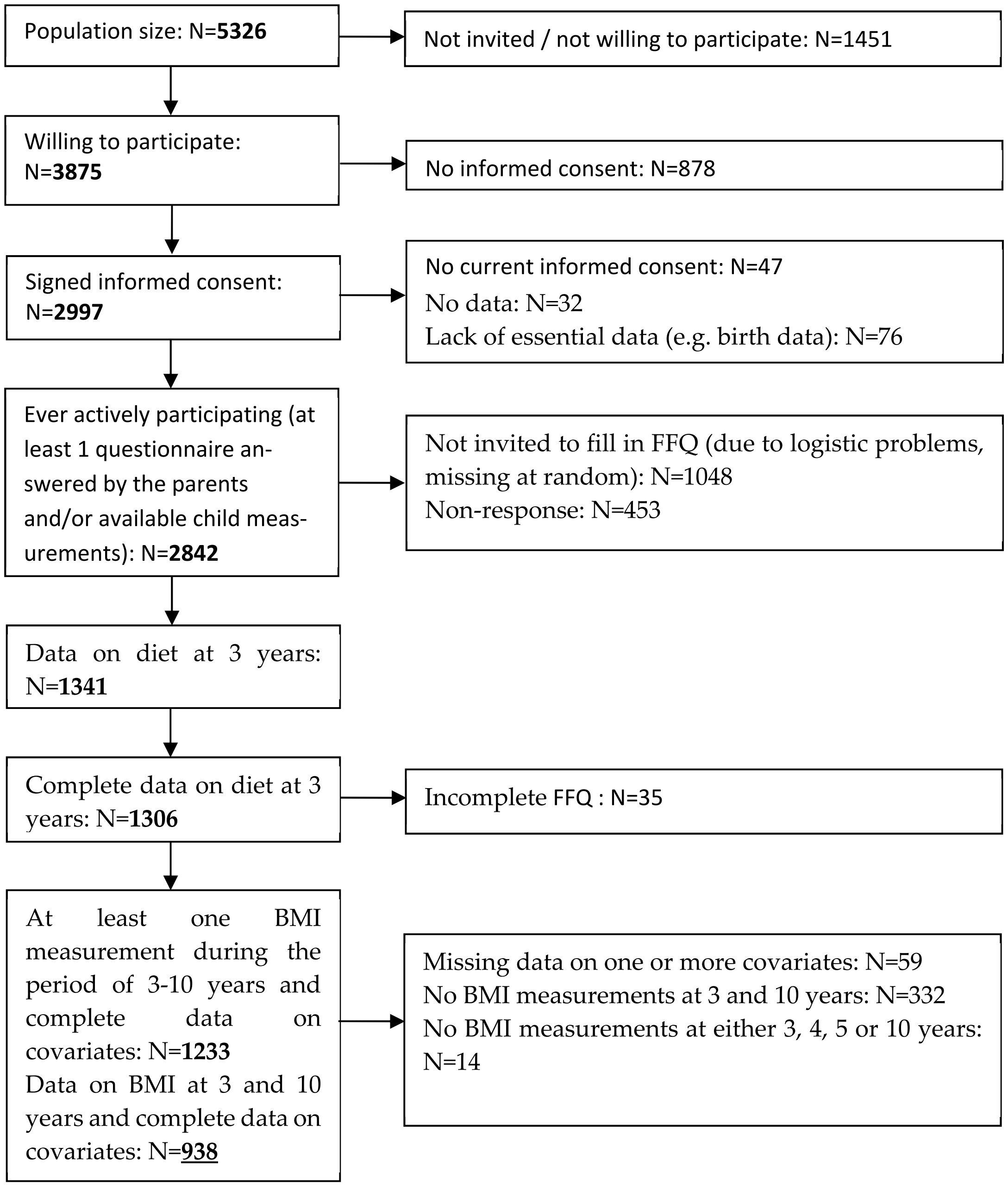

Supplementary Table S1. Food grouping for PCA

**Food group**	**Food items**
Water	Water and tea (without sugar)
Vegetables	Raw and cooked vegetables and beans
Fruit	Fruit and fruit compote
Whole-grain bread	Whole-grain bread, rye bread, whole-grain crackers
Fish	Fish, shellfish and fish sticks
Sauces	Ketchup, mayonnaise, brown sauce, apple sauce and other sauces
Potatoes	Plain potatoes (excluding fried or baked potatoes)
Eggs	Fried or baked potatoes and French fries
Fried and baked potatoes	Water and tea (without sugar)
Savory dishes	Composite dishes
Chicken	All processed and non-processed chicken/poultry (except chicken nuggets)
Meat	All processed and non-processed meat (except those included in 'savory snacks' group)
Milk and buttermilk	Plain milk (full-fat, semi-skimmed or skimmed) and plain sour milk
Dairy desserts	Yogurt (full-fat, semi-skimmed or skimmed yoghurt), custard, pudding and cream
Crisps	Crisps and prawn crackers
Cheese	Cheese and cream cheese
Cakes and confectionery	Cakes, pancakes, wafels, pastry, ice cream, candy and chocolate
Butter and oil	Butter, oil and margarine
White bread	White bread, toast, currant bread, baguette, croissant
Breakfast cereals	Cornflakes, breakfast cereals and muesli

Added sugar	Sugar, honey or syrup added to foods and drinks
Sweet bread toppings	Jam, chocolate spreads, peanut butter, sprinkles
Sugar-sweetened beverages	Soft drinks, fruit drinks and lemonade with sugar
Cookies	Cookies, biscuits and muesli bars
Rice and pasta	Rice and pasta
Vegetarian meat substitutes	Meat substitutes made of soy, quorn or tahoe
Porridge	Oatmeal and wheat porridge
Soya milk products	Soy milk, soy dessert, flavoured soy milk
Nuts and raisins	Peanuts, nuts and raisins
Crackers	Waffels, rusks, crackers and soup sticks
Savory snacks	Pizza, hamburgers, sausage rolls, chicken nuggets, meat croquettes and hot dogs
Dairy drinks with sugar	Yoghurt drinks with sugar and chocolate-flavored milk
Light drinks	Sugar-free, artificially sweetened flavored drinks

Supplementary Table S2. Associations between dietary patterns and overweight at 3 years, sensitivity analysis using the WHO definition of overweight (N=938).

	Overweight at 3 years*					
	Model 1, crude			Model 2, adjusted		
	OR	95% CI	p-value	OR	95% CI	p-value
Dietary pattern						
Pattern 1: 'minimally processed foods foods'	0.77	0.53, 1.12	0.17	0.78	0.52, 1.15	0.20
Pattern 2: 'ultra-processed foods'	1.47	1.03, 2.10	0.04	1.36	0.92, 2.02	0.12

* defined as BMI-sds according to. Both pattern scores were used as determinants simultaneously, in order to adjust for the adherence for the other pattern. Model 2 is adjusted for maternal age, pre-pregnancy BMI, parity, ethnicity, maternal smoking during pregnancy, educational level, birth weight and gestational age.

Supplementary Table S3. Associations between dietary patterns and overweight at 10 years, sensitivity analysis using the WHO definition of overweight (N=938).

	Overweight at 10 years*					
	Model 1, crude			Model 2, adjusted		
	OR	95% CI	p-value	OR	95% CI	p-value
Dietary pattern						
Pattern 1: 'minimally processed foods foods'	1.02	0.88, 1.19	0.77	1.06	0.90, 1.25	0.49
Pattern 2: 'ultra-processed foods'	1.28	1.10, 1.50	<0.01	1.21	1.02, 1.44	0.03

* defined as BMI-sds. Both pattern scores were used as determinants simultaneously, in order to adjust for the adherence for the other pattern. Model 2 is adjusted for maternal age, pre-pregnancy BMI, parity, ethnicity, maternal smoking during pregnancy, educational level, birth weight and gestational age.

Supplementary Table S4. Mean differences in estimated BMI-sds for quartile of adherence (Q1 vs. Q4) to the dietary patterns at each age, derived from the random effects linear regression model (N=1233).

	Pattern 1: 'minimally processed foods'		Pattern 2: 'ultra-processed foods'	
	Mean difference (95% CI)	p-value	Mean difference (95% CI)	p-value
Age (years)				
3	-0.04 (-0.11, 0.03)	0.25	0.04 (-0.04, 0.12)	0.31
4	-0.09 (-0.16, -0.01)	0.02	0.00 (-0.09, 0.08)	0.92
5	-0.03 (-0.10, 0.03)	0.33	-0.06 (-0.14, 0.02)	0.15
10	-0.08 (-0.16, -0.01)	0.03	-0.15 (-0.23, -0.06)	0.001

Both pattern scores were used as determinants simultaneously, in order to adjust for the adherence for the other pattern. Model 2 is adjusted for maternal age, pre-pregnancy BMI, parity, ethnicity, maternal smoking during pregnancy, educational level, birth weight and gestational age.

Supplementary Table S5. Associations between dietary patterns and BMI-sds development between 3 and 10 years of age (N=1233).

	B	95% CI	p-value
Pattern 1: 'minimally processed foods'	0.03	-0.02, 0.09	0.26
Pattern 2: 'ultra-processed foods'	-0.03	-0.09, 0.03	0.31
Age (years)			
4	0.06	0.02, 0.10	<0.01
5	0.39	0.35, 0.44	<0.001
10	0.30	0.24, 0.36	<0.001
Age x Pattern 1: 'minimally processed foods'			
4	0.04	0.00, 0.08	0.06
5	-0.01	-0.05, 0.03	0.78
10	0.04	-0.02, 0.10	0.22
Age x Pattern 2: 'ultra-processed foods'			
4	0.04	0.00, 0.07	0.07
5	0.08	0.03, 0.12	<0.001
10	0.15	0.08, 0.21	<0.001

Both pattern scores were used as determinants simultaneously, in order to adjust for the adherence for the other pattern. Model is adjusted for maternal age, pre-pregnancy BMI, parity, ethnicity, maternal smoking during pregnancy, educational level, birth weight and gestational age.
